# Editorial for the Special Issue on MEMS/NEMS Devices and Applications, 2nd Edition

**DOI:** 10.3390/mi16020189

**Published:** 2025-02-07

**Authors:** Yao-Chuan Tsai, Pin-Chun Huang, Ching-Liang Dai

**Affiliations:** 1 Department of Bio-Industrial Mechatronics Engineering, National Chung Hsing University, Taichung 402, Taiwan; yctsaii@dragon.nchu.edu.tw; 2 Department of Mechanical and Aerospace Engineering, University of California, Irvine, Engineering Gateway 4200, Irvine, CA 92697, USA; pinchuh1@uci.edu; 3 Department of Mechanical Engineering, National Chung Hsing University, Taichung 402, Taiwan

## 1. Introduction

Microelectromechanical systems (MEMSs) and nanoelectromechanical systems (NEMSs) are revolutionary technologies that merge mechanical and electronic components on microscopic and nanoscopic scales [1,2,3]. MEMSs operate at the microscale, while NEMSs represent the next evolution, with components functioning at the nanoscale. Together, these technologies form the backbone of numerous modern devices, driving innovation across industries ranging from healthcare to consumer electronics. MEMSs and NEMSs integrate mechanical elements, sensors, actuators, and electronics on a single silicon chip [4,5,6]. MEMS devices leverage microfabrication techniques derived from the semiconductor industry, including photolithography, etching, and deposition. Components such as micro-scale gears, levers, and membranes operate in conjunction with electronic circuits, enabling complex functionalities. NEMSs, on the other hand, build upon MEMS principles but operate at the nanoscale, utilizing materials like graphene, carbon nanotubes, and nanowires [7,8,9]. The smaller size of NEMSs offers advantages such as faster response times, reduced energy consumption, and the ability to detect extremely subtle forces or changes in the environment.

**Figure 1:**
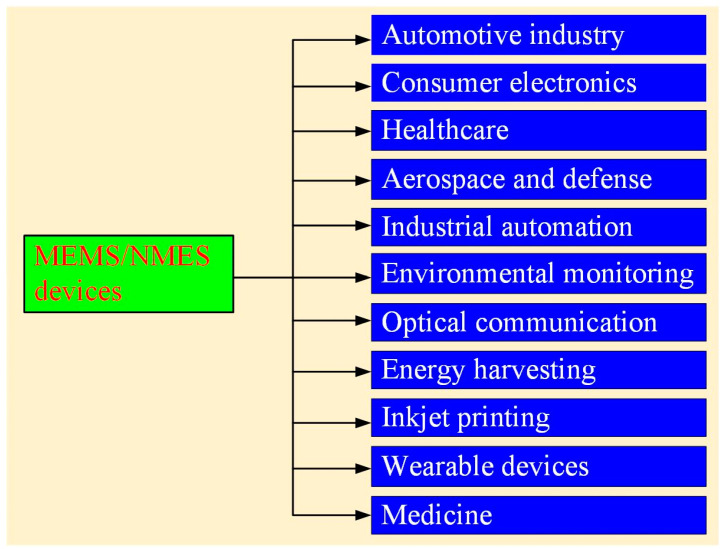
Applications of MEMS/NEMS devices.

As shown in Figure 1, MEMS devices are applied in numerous fields. In the automotive industry, they serve as accelerometers for airbags and gyroscopes for stability control systems [10]. In healthcare, MEMSs enable innovations such as lab-on-a-chip devices for diagnostics, drug delivery systems, and biosensors for glucose monitoring [11,12]. MEMS-based microfluidic chips are also used for deoxyribonucleic acid (DNA) analysis and point-of-care testing. In consumer electronics, MEMS devices are integral to smartphones, tablets, and gaming consoles [13]. Applications include motion sensing for screen orientation, vibration motors, microphones, and optical image stabilization in cameras. In aerospace and defense, MEMS gyroscopes and accelerometers are critical in navigation systems for drones, aircraft, and missiles [14]. They provide precise motion and orientation data for stable flight and accurate targeting. In industrial automation, MEMS sensors monitor force, displacement, and vibration in industrial machinery [15]. These devices are essential for predictive maintenance and improving operational efficiency in manufacturing processes. In environmental monitoring, MEMS devices are used in air and water quality sensors to detect pollutants and measure environmental parameters such as humidity, temperature, and gas concentration [16,17]. These are vital for environmental protection and smart city initiatives. In optical communications, MEMS-based optical switches and mirrors play a key role in telecommunication networks [18,19,20]. They enable efficient routing of light signals in fiber optic communications, reducing power consumption and enhancing speed. In energy harvesting, MEMS devices, such as thermoelectric energy harvesters, capture mechanical heat and convert it into electrical energy [21,22]. These are used in internet of things (IoT) devices for self-powered sensors. In inkjet printing, MEMS nozzles in inkjet printers enable precise ejection of ink droplets, ensuring high-resolution printing [23]. This application is also extended to 3D printing technologies. In wearable devices, MEMS accelerometers, gyroscopes, and pressure sensors are core components of wearable fitness trackers, smartwatches, and augmented reality glasses [24]. These devices track motion, monitor vital signs, and enhance user interaction. NEMS devices excel in applications requiring extreme sensitivity and precision. In medicine, NEMS devices are utilized to detect biomolecules, such as proteins, DNA, and disease markers, with exceptional sensitivity. These biosensors enable early diagnosis of diseases like cancer or infectious diseases by identifying trace quantities of specific biomarkers [25]. In scientific research, NEMS resonators are used for studying quantum phenomena. Additionally, NEMS are pivotal in next-generation data storage and computing technologies due to their ultra-low power requirements and high-speed operation. As advancements in fabrication and integration continue, these systems will remain critical to the development of smarter, smaller, and more efficient technologies across diverse fields.

This Special Issue has compiled numerous outstanding studies on NEMS/MEMS devices and their applications. These include a wide range of sensors [26,27,28,29,30], actuators [31,32,33,34], energy harvesters [35], and antennas [36].

## 2. MEMS/NEMS Sensors in This Special Issue

This Special Issue includes many studies on MEMS/NEMS sensors. Their key features and highlights are introduced below. Wang et al. [26] present the development and evaluation of a piezoelectric pressure transducer utilizing polyvinylidene fluoride (PVDF) for investigating unsteady phenomena in supersonic shock wave/boundary layer interactions. Unlike traditional sensors such as MEMS-based devices or pressure-sensitive paint, this research introduces a low-cost, flexible, and lightweight solution that enhances both sensitivity and adaptability. The PVDF sensor array, designed specifically for capturing low-frequency pressure fluctuations, features a unique structure fabricated via a double-sided screen-printing technique. The array comprises 110 µm-thick PVDF films with printed silver electrodes, arranged in a 3 × 6 configuration. The researchers employed finite element simulations to optimize the design, ensuring minimal signal crosstalk and stable performance up to 300 kHz. Experimental calibration and validation were conducted using a custom acoustic setup and wind tunnel tests at Mach 2, confirming the sensor’s capability to measure dynamic pressure changes effectively. Compared to conventional Kulite sensors, the PVDF array demonstrated competitive sensitivity while being significantly more flexible and scalable. Additionally, the modular assembly of the sensing and circuit components addressed challenges like signal attenuation and crosstalk. This innovative approach highlights the potential of PVDF sensors in capturing complex aerodynamic behaviors, providing a promising alternative for advanced research in high-speed flows and low-frequency unsteady phenomena.

Lv et al. [27] study the design and optimization of a MEMS resonant pressure sensor to achieve a wide measurement range and high sensitivity, addressing the challenges in traditional sensor designs. A novel vertical resonator structure is introduced, with the resonant beam aligned perpendicularly to the sensitive diaphragm. This configuration improves diaphragm rigidity without increasing its thickness, enabling a broader range while maintaining sensitivity. To optimize the structural parameters, the study employs a combination of back propagation neural networks and the non-dominated sorting genetic algorithm II. These methods ensure the sensor’s performance by resolving modal interference issues and enhancing linearity. The core materials include silicon-based components for the diaphragm and resonator, while the fabrication process integrates micro-scale engineering techniques. The optimized sensor achieves a sensitivity of 4.23 Hz/kPa over a range of 1–10 MPa, with a linear influence factor of 38.07—significantly outperforming conventional designs. This work represents high-performance MEMS pressure sensors, with potential applications in aerospace, marine exploration, and other industries requiring precise pressure measurements.

Zhou et al. [28] present the design of a quad mass gyroscope (QMG) integrating a compliant mechanical amplification structure to significantly improve sensitivity and performance. The research introduces a rhombus-shaped V-spring system for mechanical motion amplification, amplifying the Coriolis force before it is processed electronically, thereby reducing the noise interference and enhancing the signal clarity. The gyroscope is fabricated using silicon-on-insulator (SOI) and gold–silicon eutectic bonding techniques, featuring a compact 4 × 5 mm structure. The QMG’s design incorporates four symmetrically arranged proof masses connected by U-shaped and V-shaped springs, which facilitate mechanical amplification and decouple drive and sense modes. Finite element analyses validated the structural integrity and performance, with the gyroscope achieving a resonant frequency of 11.67 kHz and a mechanical amplification factor of 3.65 times. Testing demonstrated a scale factor nonlinearity of 54.69 ppm over a ±400°/s range and a bias stability of 44.53°/h. This work advances gyroscope technology by integrating mechanical amplification at the front end, offering enhanced sensitivity and robustness against external vibrations, making it suitable for high-performance inertial sensing applications.

Xu et al. [29] explore the development of a microscale gas chromatography (µGC) system based on a novel multi-sensing progressive cellular architecture. The research aims to optimize the detection and analysis of volatile organic compounds (VOCs) through a compact, efficient, and highly integrated design. The innovative aspect of the study lies in its use of multithreaded control software for managing the complex interplay of various hardware components in real-time, ensuring enhanced performance and flexibility. The µGC system comprises three micro-gas chromatography cells, each tailored to specific ranges of analytic volatility. These cells incorporate preconcentrators, separation columns, and three complementary detectors, leveraging diverse sensing principles, including capacitive and photoionization detection. The device uses silicon-based microfabrication with precise heater and sensor integration for temperature and flow control. Fabrication includes advanced photolithography and etching techniques for component miniaturization and integration. The control software, implemented on a Raspberry Pi platform, enables the concurrent operation of pumps, valves, heaters, and sensors via a multithreading architecture. This approach facilitates precise synchronization and efficient resource utilization, achieving minimal timing errors and robust system reliability. Experimentation demonstrates the system’s capability to analyze 18 VOCs with high sensitivity and reproducibility, supported by a flexible graphical user interface for real-time monitoring and configuration. This work highlights the potential of µGC systems in environmental monitoring, industrial applications, and portable chemical analyses, establishing the foundation for future innovations in integrated microscale systems.

Song et al. [30] focus on designing and optimizing a milling force measurement tool system embedded with thin-film strain sensors. The research addresses the challenge of accurately measuring milling forces by employing an innovative double-end-supported thin-film strain sensor. This novel design enhances sensitivity and strain measurement reliability through the integration of a resistance grid beam on the transition layer. The sensors comprise multiple layers: a stainless-steel substrate, a TiN transition layer for improved binding, a Si_3_N_4_ insulating layer, and a Ni_80_Cr_20_ resistance grid. Advanced microfabrication techniques, including magnetron sputtering, photolithography, ion beam etching, and wet etching, were utilized to create the sensors. The structural optimization process introduced features like H-type and arc-type substrates to minimize stress concentrations and improve mechanical performance. Experimentally, the system demonstrated a resistive sensitivity coefficient increased by 20%, achieving 51.2% compared to conventional designs. The results were validated using various characterization tools, including confocal and atomic force microscopy, revealing high precision in the sensor’s microstructure. This research represents a significant advancement in sensor technology, enabling precise real-time force measurement in milling processes, with potential applications in manufacturing and machining industries.

## 3. MEMS/NEMS Actuators in This Special Issue

This Special Issue also features many studies on MEMS/NEMS actuators. The features and highlights of these actuators are described below. Shuaibu et al. [31] introduce a laterally actuated silicon-to-silicon direct current MEMS switch designed for power switching in high-voltage and harsh environments. The research highlights the innovation of employing a chevron-shaped electrothermal actuator that uses an aluminum heater layer for actuation, combined with a silicon dioxide insulation layer to ensure electrical isolation between the heater and transmission line. This design overcomes the limitations seen in traditional MEMS switches, such as high contact resistance and material degradation. The switch is fabricated using the PiezoMUMPs process [31], involving multi-layered structures with precise alignment of silicon, aluminum, and silicon dioxide. The actuator achieves an in-plane displacement of 2.52 µm to close the contact gap at an actuation voltage of 1.2 V and a current of 205 mA, with a switching speed below 5 ms. Rigorous simulations and testing confirmed the switch’s capability to withstand a breakdown voltage of up to 350 V, with low contact resistance measured at 150 Ω. Innovative techniques, such as controlling the etching angle and optimizing the thermal actuator’s design, mitigate challenges such as out-of-plane deformation and contact misalignment. Additionally, the integration of narrow silicon beams reduces thermal gradients, enhancing stability and performance. This work significantly advances the field by providing a robust and reliable MEMS switch solution for high-voltage applications.

Wang et al. [32] explore strain-induced frequency splitting in parity-time (PT) symmetric coupled silicon resonators. It innovatively leverages the unique properties of PT symmetry, which involves balancing gain and loss within coupled resonators, to enhance sensitivity to strain near the exceptional point. This study integrates theoretical modeling, finite element simulations, and experimental validations. The resonators, constructed using a SOI process, employ precise feedback circuits to achieve the required negative damping for PT symmetry. Key components include silicon beams of 25 µm thickness, 400 µm substrates, and a gap of 2 µm. The fabrication process involves deep reactive-ion etching and gold layer patterning. Experimental results confirm the system’s heightened strain sensitivity, achieving significant frequency splitting under applied strain. This work broadens PT symmetry applications and sets the foundation for advanced sensing technologies.

Chimerad et al. [33] present an innovative bio-inspired soft actuator designed to autonomously remove copper ions from aqueous environments. This miniaturized device integrates fuel-free, self-propelling capabilities utilizing the Marangoni effect, mimicking the propulsion mechanisms of semi-aquatic beetles. The actuator’s unique design and functionality represent a significant advancement over traditional stationary hydrogels used for heavy metal remediation, addressing mobility challenges in uneven contamination scenarios. The device’s structure consists of a hydrogel fabricated from acrylamide derivatives and cross-linked with polyethylene glycol diacrylate. A gear-shaped mold, created using computer-aided design and 3D printing, facilitates its rotational movement and increases its surface area for absorption. The manufacturing process incorporates ultraviolet polymerization and freeze-drying, yielding a porous material capable of dynamic environmental responses. Through surface tension gradients induced by hydrophilic and hydrophobic surface transitions, the actuator achieves autonomous locomotion, exposing fresh hydrogel surfaces for continuous copper ion absorption. Experimental results reveal its capacity to operate effectively in copper-ion concentrations ranging from 500 to 3000 ppm, with absorption efficiency validated through scanning electron microscope, Fourier-transform infrared spectroscopy, and colorimetric analysis. The actuator demonstrated scalability and adaptability, autonomously covering large water surface areas without external energy sources. This study marks a breakthrough in active, eco-friendly water purification technologies, paving the way for versatile applications in environmental remediation.

Shan et al. [34] focuses on developing and analyzing a vertical comb-drive actuator for MEMS micromirrors, aimed at achieving enhanced stability and precise control during optical scanning. It introduces a self-alignment fabrication technique that ensures a height difference between movable and fixed comb structures, reducing alignment errors and improving reliability. This actuator leverages the quasi-static driving principle to address instability issues such as lateral bending, displacement, and rotational failures of comb fingers. The micromirror’s structural components include a SOI substrate and torsion beams, with a 0.8 mm mirror diameter and 30 µm device layer thickness. The height difference between comb structures is achieved through precise dry etching and photolithography processes. The actuator demonstrates stable operation below 60 V, and reaches a static mechanical deflection angle of 2.25° at 60 V, aligning closely with the theoretical models. Critical failure modes were observed at higher voltages, validating the proposed instability models. This research offers a robust foundation for designing reliable MEMS micromirrors with potential applications in optical communications and autonomous systems.

## 4. MEMS/NEMS Energy Harvesters and Antennas in This Special Issue

This Special Issue features a study on a MEMS energy harvester, with its key features described below. Chen et al. [35] explore the fabrication and performance evaluation of a photovoltaic microgenerator created using the complementary metal oxide semiconductor (CMOS)-MEMS process, aiming to achieve efficient energy conversion within a compact structure. The research emphasizes innovative modifications, such as a mesh-patterned p–n junction design and the post-fabrication removal of the silicon dioxide layer, which enhance light absorption and energy conversion efficiency. The microgenerator is constructed using silicon-based materials, featuring patterned p-type and n-type doping layers that optimize charge separation and minimize leakage currents. The fabrication process employs the standardized 0.18 µm CMOS process, followed by a buffered oxide etching step to expose the p–n junctions, enabling direct illumination and improved power generation capabilities. Simulations and experimental validation demonstrate the device’s effectiveness, achieving an energy-conversion efficiency of 12.5% under an irradiance of 1000 W/m^2^. It records an open-circuit voltage of 0.53 V, a short-circuit current of 233 µA, and a maximum output power of 99 µW within a compact area of 0.79 mm^2^. This study provides a significant step forward in the development of scalable, efficient photovoltaic microgenerators, suitable for powering IoT devices, sensors, and other compact electronics.

Li et al. [36] investigate a magnetoelectric thin-film antenna, focusing on optimizing its structure to enhance bandwidth and radiation performance. This study addresses the challenges of narrow operating bandwidths and low antenna gains inherent in traditional magnetoelectric antennas. By analyzing the interplay between structural dimensions and electromagnetic properties, the research introduces a ring-shaped design with variable inner radii, leveraging stress concentration effects to improve performance. The core of the antenna comprises piezoelectric AlN and magnetostrictive FeGaB layers, fabricated using a thin-film bulk-acoustic resonator approach. Through COMSOL [36] multiphysics simulations, the team analyzed resonance behavior, stress distribution, and radiation patterns, revealing that an inner radius of 50 µm maximized bandwidth by 104% and enhanced radiation efficiency. The study also validated its findings against experimental data from previous designs, ensuring reliability. This research introduces a scalable optimization model that correlates stress distribution with radiation capability, advancing magnetoelectric antenna applications in compact communication systems operating at lower frequencies.
